# White spot syndrome virus VP28 specific double-stranded RNA provides protection through a highly focused siRNA population

**DOI:** 10.1038/s41598-017-01181-w

**Published:** 2017-04-21

**Authors:** Pål Nilsen, Marius Karlsen, Kallaya Sritunyalucksana, Siripong Thitamadee

**Affiliations:** 1 PHARMAQ AS, PO Box 267, N-0213 Oslo, Norway; 2 Center of Excellence for Shrimp Molecular biology and Biotechnology (Centex Shrimp), Faculty of Science, Mahidol University, Bangkok, 10400 Thailand; 3 Department of Biotechnology, Faculty of Science, Mahidol University, Bangkok, 10400 Thailand; 4 Shrimp-Pathogen Interaction (SPI) Laboratory, National Center for Genetic Engineering and Biotechnology (BIOTEC), National Science and Technology Development Agency (NSTDA), Yothi Office, Rama VI Rd., Bangkok, 10400 Thailand

## Abstract

Several studies have demonstrated that injection of double-stranded RNAs (dsRNA) homologous to mRNA for the white spot syndrome virus (WSSV) viral protein 28 (VP28) can induce protection in shrimp against WSSV through RNA interference (RNAi). In comparison to shrimp injected with either PBS or a green fluorescent protein (GFP) nonspecific dsRNA, we obtained nearly complete protection against WSSV infection in shrimp injected with VP28 dsRNA. Upregulation of host genes associated with small RNA silencing was measured 48 hours post treatment in groups injected with dsRNA, and although the VP28-treated group remained moderately upregulated after challenge with WSSV, many-fold higher induction was observed in both control groups reflecting the ongoing viral infection. RNA sequencing of VP28-treated shrimp demonstrated a siRNA population dominated by high levels of 22 nt long molecules narrowly targeting the VP28 mRNA both before and after challenge with WSSV. Conversely, while no siRNAs targeting WSSV were detected before challenge, a broad response of 22 nt siRNAs mapping across the entire WSSV genome were found in both control groups after challenge. These results give detailed insight to how dsRNA targeting VP28 function to induce protection against WSSV, by generating a highly focused population of 22 nt long siRNA molecules.

## Introduction

As aquaculture production of shrimp has grown into the most valuable seafood commodity over the last 30 years^1^, disease outbreaks have become an increasing challenge for the industry. Viruses have made a large impact on growth and survival of shrimp in artificial culture systems^2^, and white spot disease (WSD) caused by WSSV^3^, a large DNA virus with a 300 kb double-stranded genome in the family *Nimaviridae*, has alone caused an estimated accumulated loss of $8–$15 billion in the period 1992–2012^4^.

Shrimp can be effectively protected against WSSV and other viruses by injecting specific dsRNA targeting viral genes^5^. WSSV VP28, an envelope protein suggestively involved in endosome escape through its interaction with host Rab7^6, 7^, has been identified as a potent target for dsRNA treatment in comparative studies^8–10^.

In general, the concept of small RNA silencing involves Dicer (Dcr) endonuclease enzymes that recognize and cleave different variants of RNAs with double-stranded motifs, feeding various regulatory pathways with short duplex RNAs. The siRNA pathways play an important role in somatic cells detecting long dsRNA molecules from viral- or transposable element replication. Duplex siRNA molecules are loaded into the RNA-induced silencing complex (RISC). In RISC, the passenger- and guide strands are separated, and the guide strand remains attached to be utilized in detection and, aided by Argonaute (Ago) protein’s effector mechanism in RISC, cleavage of mRNA transcripts with complementarity to the siRNA sequence. Cleavage of mRNA transcripts by the action of siRNA and RISC inhibits translation of proteins encoded by viruses and mobile genetic elements, and is referred to as RNA interference^11–13^.

As for *Drosophila* ^14–16^, *P. vannamei* has been found to encode two variants of Dicer and Argonaute, and among these LvDcr2 and LvAgo2 are employed by the siRNA pathway^17–19^. Although Dcr1 and Ago1 are normally associated with the miRNA pathway in *Drosophila* ^14, 20^, a possible involvement in the antiviral response has been shown for homologue genes in white leg shrimp^21–23^. Contrary to the direct involvement of Dicer and Argonaut in RNAi, Sid-1 was described in *Caenorhabditis elegans* as a transmembrane dsRNA transport protein localized to the plasma membrane and required for the spread of a systemic RNAi signal^24, 25^. A Sid-1 homologue, LvSid-1 was later discovered in *P. vannamei* and found to be specifically upregulated in the presence of dsRNA^19^, and thus potentially indirectly involved in an antiviral RNAi response.

In *Drosophila*, dsRNA is cleaved by Dicer-2 into siRNA 21–23 nucleotides in length^26^, although the most effective siRNA duplexes for this organism has been found to consist of 21-nucleotide siRNAs with 2-nucleotide 3′ overhangs^27^. Recently, an RNA sequencing study on WSSV infected shrimp (*Penaeus chinensis*) suggested a siRNA population to be within the 21–23 nucleotide range with a peak at 22 nucleotides^28^, and a similar study on the bumble bee (*Bombus terrestris*) also emphasizes on a 22 nucleotide siRNA peak^29^.

Although it is clear that shrimp can be effectively protected against WSSV through the activation of RNAi, the RNAi response to treatment has not been thoroughly characterized. Here we demonstrate how the highly protective RNAi response in shrimp treated with WSSV VP28 specific dsRNA differs from control shrimp receiving either nonspecific GFP dsRNA or no treatment through a PBS injection. In response to a WSSV VP28 specific dsRNA treatment, a relatively modest induction of RNAi-associated genes was followed by a highly intensive and focused siRNA population dominated by molecules of 22 nt in length. This population remained unchanged after challenge with WSSV, in contrast to the two unprotected control groups where a population of siRNAs globally targeting the WSSV genome developed.

## Results

### Detection of dsRNA in treated shrimp

**Figure 1:**
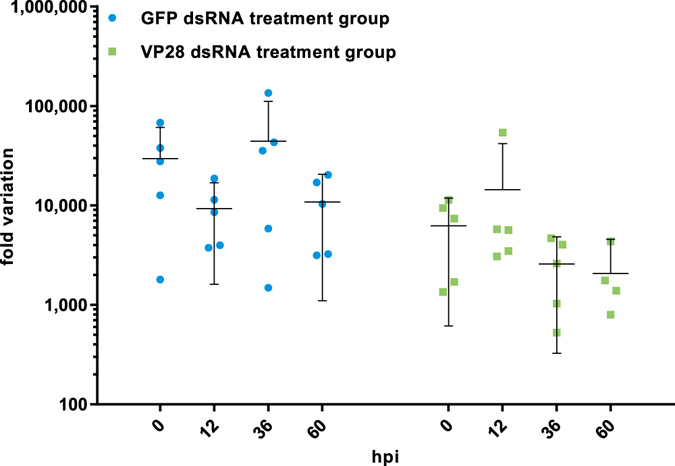
Detection of injected dsRNA in experimental animals. Shrimp (2.618 g ± 0.527 SD, n = 30) injected with 42.85 µg dsRNA were sampled (n = 5) at indicated time points, and dsRNA levels were measured by RT-qPCR using assays with specificity for the injected treatments. Another experiment verified that when using primers nonspecific for the VP28 dsRNA treatment, but with specificity for VP28 mRNA, no Cq-values were obtained through 40 cycles for shrimp in the VP28 dsRNA treatment group. One-way ANOVA did not reveal any significant changes between groups or time points at α = 0.05 (p = 0.255). Error bars indicate the 95% CI around the mean.

Groups consisting of 5 separate tanks each holding 20 shrimp were injected intramuscularly (IM) with either PBS or dsRNA homologous to the GFP-specific or VP28-coding sequence. RT-qPCR was performed on shrimp stomach tissue in order to verify that both the delivered dsRNA targeting GFP and VP28 were present in the treated shrimp during the sampling period. Both dsRNA constructs were detected 48 hours after treatment and onwards throughout the sampling period (Fig. 1). No significant changes in dsRNA level over time could be detected by one-way ANOVA (p = 0.255), indicating a stable presence of these dsRNAs up until 60 hours post infection (hpi).

### VP28 dsRNA treatment provides nearly full protection against WSD in *P. vannamei*

**Figure 2:**
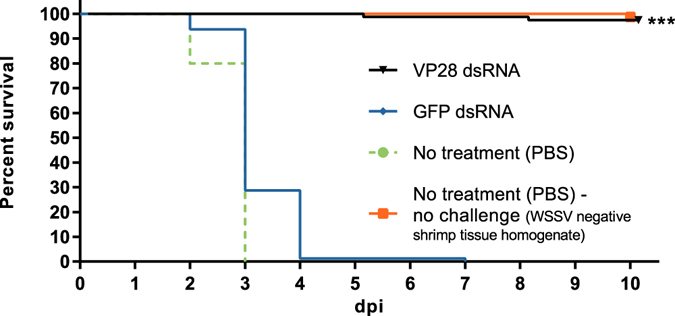
Efficacy of IM dsRNA treatment measured by survival percentage after IM WSSV challenge. 48 hours post treatment administration, 100 shrimp in 5 parallel tanks per treatment group were IM challenged with a WSSV dose equivalent to approximately 1000 WSSV genome copies per shrimp. For each group, 4 tanks holding 20 shrimp each were monitored through 10 dpi to assess the efficacy of VP28 dsRNA treatment, and relative survival from these shrimp is here presented in a Kaplan-Meier plot. Log-rank (Mantel-Cox) test with Bonferroni correction (α = 0.05/21) was performed on VP28 dsRNA treatment group in comparison to the GFP dsRNA treatment group (p < 0.0001).

**Figure 3:**
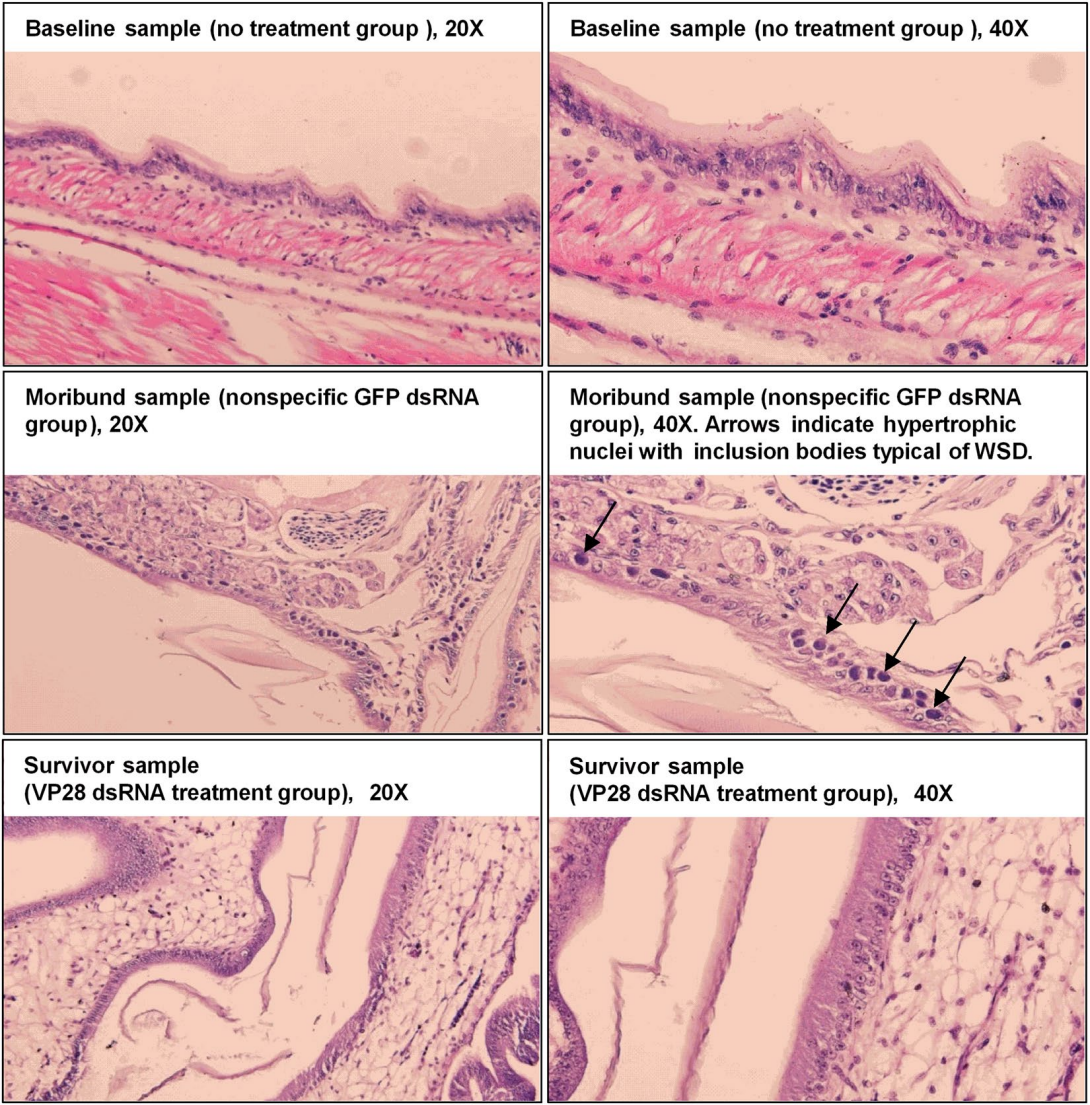
Histology examination carried out to identify WSSV-specific pathology in experimental animals. Shrimp collected at different time points were sectioned and stained with hematoxylin-eosin for histological examination. All baseline samples were collected from the no treatment (PBS) group prior to challenge at 0 dpi, and all survivor samples were collected at 10 dpi from the VP28 dsRNA treatment group. Moribund shrimp were collected from the GFP dsRNA treatment group during the challenge trial, where infected cells exhibiting hypertrophic nuclei containing eosinophilic inclusion bodies were identified (indicated by black arrows).

**Figure 4:**
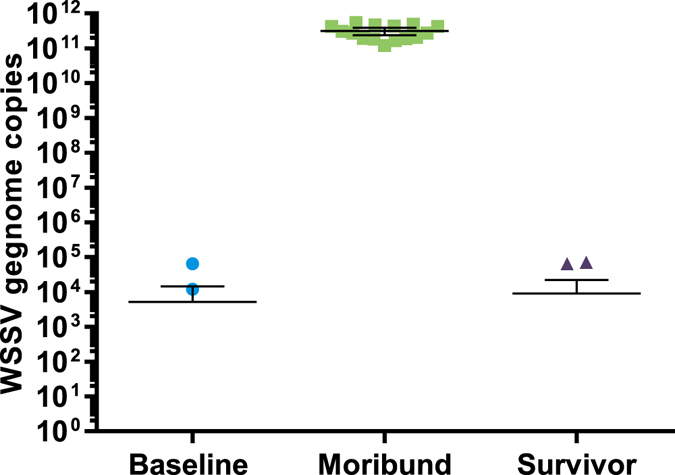
qPCR detection of WSSV in experimental animals. DNA isolated from uropods from shrimp collected at different time points before, during and after challenge was subjected to a qPCR assay detecting WSSV VP28. Cq-values from individual shrimp were compared to a standard curve, made up of 10-fold serial dilutions of a plasmid containing a VP28 insertion, to obtain absolute quantities of WSSV genome copy numbers. The Y-axis represents the absolute WSSV genome copy numbers obtained from each individual shrimp sample. Error bars indicate the 95% CI around the mean.

The treated groups were challenged by IM injection of live WSSV 48 hours post treatment. During an observation period of 10 days, none of the challenged shrimp treated with either PBS or GFP dsRNA survived, while survival in the group treated with VP28 dsRNA was 78 of 80 shrimp (Fig. 2). Histological analysis of 8 baseline samples (no treatment (PBS), before challenge), 7 moribund samples (from nonspecific GFP dsRNA group) and 10 survivor samples (from VP28 dsRNA group) confirmed presence of histopathology compatible with WSD in all moribund shrimp, but not in baseline or survivor shrimp (Fig. 3). Absolute quantification of WSSV genomic DNA from 15 baseline samples at 0 days post infection (dpi), 15 moribund samples collected during challenge and 15 survivor samples at 10 dpi demonstrated high amounts of WSSV genomic DNA in all moribund shrimp and only trace amounts in 2 of 15 survivor shrimp (Fig. 4). Baseline samples collected prior to challenge also contained trace amounts of WSSV DNA in two of 15 shrimp, suggesting such low levels of WSSV DNA to represent either background levels in the population, or false positives. It should be noted that all baseline- and survivor samples that came up as positive were in fact shown to have amplification products in only one out of three qPCR reaction wells.

### Complete lack of WSSV replication in VP28-treated shrimp

**Figure 5:**
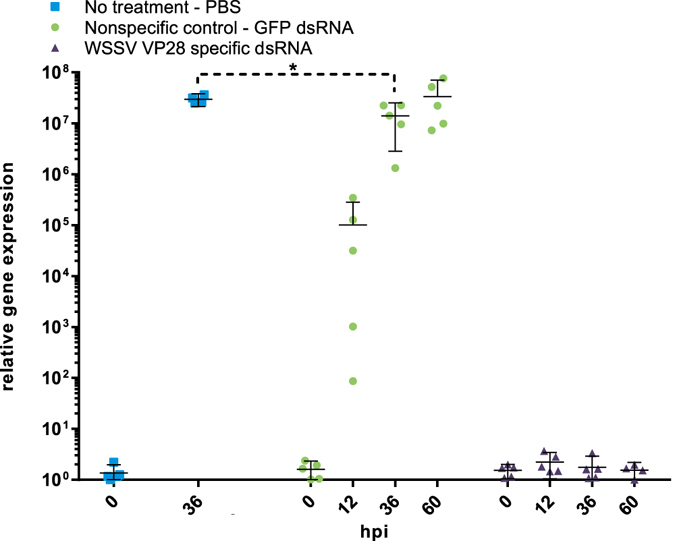
A VP28 mRNA specific RT-qPCR assay, without specificity for the VP28 dsRNA treatment, was applied in order to monitor WSSV replication rate during the first 60 hours of infection. The relative gene expression measured at 0 hpi arise from variation in reference gene expression and not VP28 mRNA, for which all samples collected at 0 hpi did not produce any Cq-values. Similarly, no Cq-values were obtained at 12, 36 and 60 hpi for the VP28 dsRNA treatment group. One-way ANOVA with Bonferroni’s multiple comparisons test was performed on GFP dsRNA treatment at 36 hpi in comparison to no treatment (PBS) at 36 hpi, showing significant variation at α = 0.05. Error bars indicate the 95% CI around the mean.

In order to measure the virus replication rate on group level over time after challenge, a RT-qPCR assay was designed to quantify VP28 mRNA in individual shrimp stomach tissue samples. This assay’s reverse primer was placed in the boundary between the ORF and the 3′ UTR to ensure that it would not amplify the VP28 dsRNA treatment construct. In the VP28 specific dsRNA treatment group, no cq-values were obtained during 40 PCR cycles, and in order to conduct a relative gene expression analysis these samples were given a cq-value of 40. Relative expression observed for this group arose from variation in reference gene levels (Fig. 5). Thus, none of the shrimp that had been treated with VP28 dsRNA were found to be positive, while high levels of WSSV were detected during the course of infection in both the PBS- and GFP dsRNA control groups. A higher amount of VP28 mRNA was evident in the PBS control group compared to the GFP dsRNA control group, suggesting some nonspecific protection from the GFP dsRNA.

### Induction of RNAi after dsRNA treatment administration is modest compared to upregulation during WSSV infection

**Figure 6:**
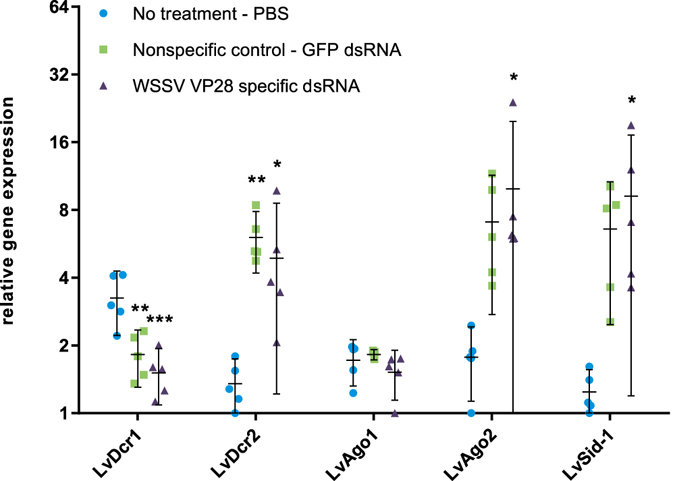
48 hours after dsRNA injection, relative gene expression on a panel of host genes associated with small RNA silencing was carried out by RT-qPCR assays. Relative gene expression was measured through fold variation from the lowest obtained normalized expression value observed in all samples. One-way ANOVA with Dunnett’s multiple comparisons test was performed individually for each gene comparing the two dsRNA treatment groups to the no treatment (PBS) group. Significant variation at α = 0.05 is indicated and error bars indicate the 95% CI around the mean.

**Figure 7:**
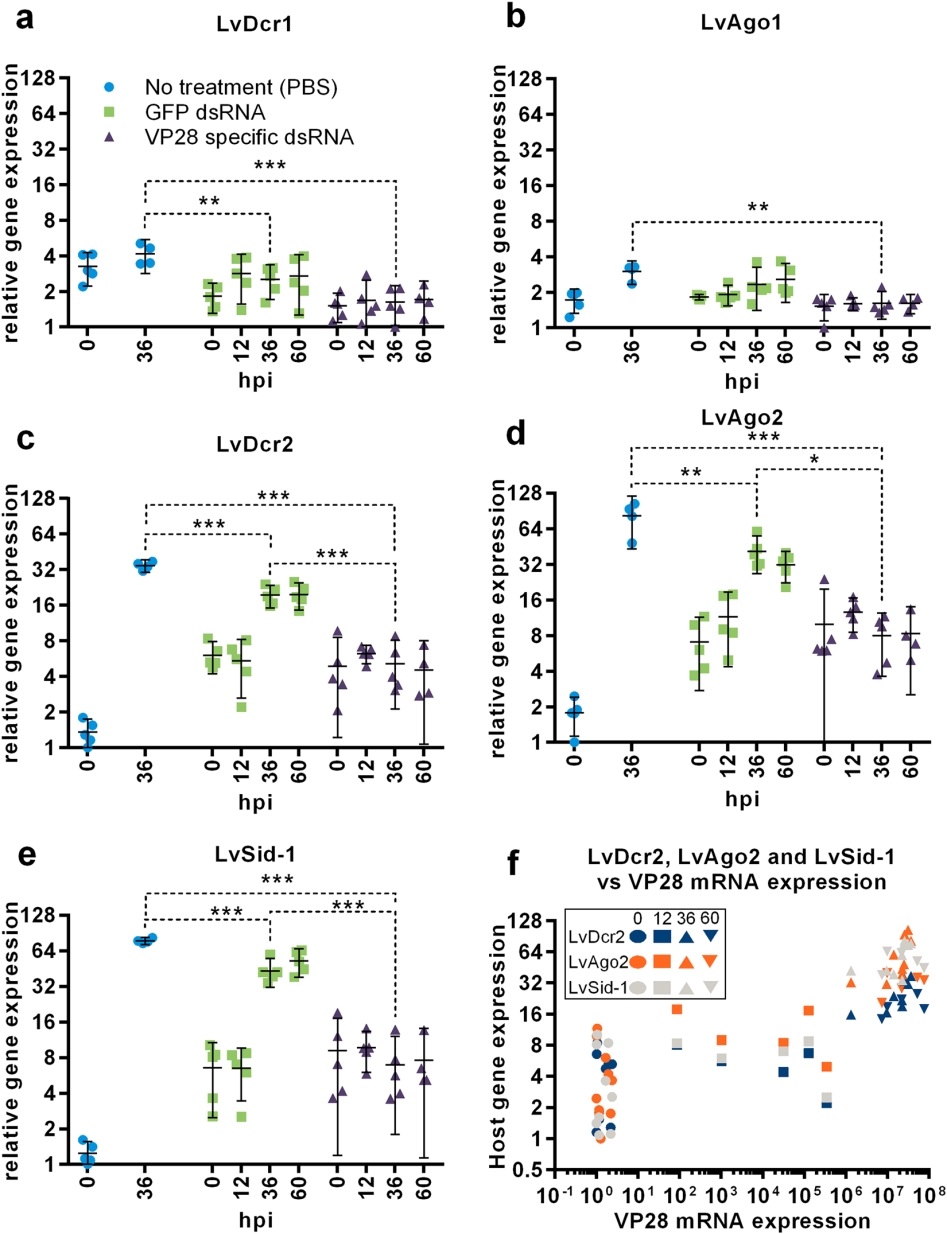
During the first 60 hours of WSSV infection, relative gene expression on a panel of host genes associated with small RNA silencing was carried out by RT-qPCR assays (**a**–**e**). Relative gene expression was measured through fold variation from the lowest obtained normalized expression value observed in all samples. One-way ANOVA with Tukey’s multiple comparisons test was performed individually for each gene comparing groups at 36 hpi. Significant variation at α = 0.05 is indicated and error bars indicate the 95% CI around the mean. To investigate LvDcr2, LvAgo2 and LvSid-1 host genes’ dependency to WSSV virus load and time since infection, all individual normalized expression values obtained for these genes in the no treatment (PBS) and GFP dsRNA treatment groups was plotted against VP28 normalized expression level registered in the respective samples (**f**).

The expression patterns of a panel of host genes associated with small RNA silencing described from *P. vannamei* were examined by RT-qPCR in samples from all groups taken 48 hours after treatment and at several time-points after challenge. Treatment with both GFP and VP28 dsRNA resulted in significant upregulation of LvDcr2, LvAgo2 and LvSid-1, but not LvDcr1, which was significantly downregulated, and LvAgo1, which remained unchanged, compared to the PBS control (Fig. 6). After challenge, LvDcr2, LvAgo2 and LvSid-1 stayed upregulated in the two dsRNA-treated groups, and also became upregulated in the PBS control group as the infection progressed (Fig. 7C–E). Expression levels of these genes were considerably higher in the PBS- and GFP dsRNA control groups compared to the VP28-treated group, where an apparently stable level was achieved. All three genes as well as viral VP28 mRNAs increased with time, but there was no strong correlation between host and viral mRNAs within each time point (Fig. 7F).

### dsRNA derived siRNAs in *P. vannamei* are predominantly 22 nucleotides long

**Figure 8:**
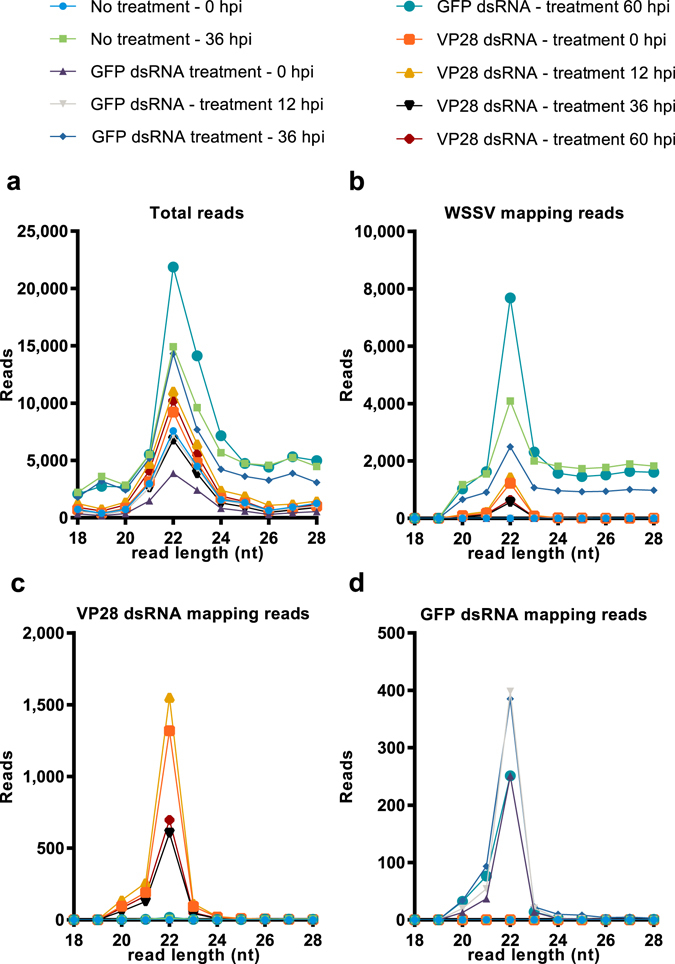
Small RNA length distribution. RNA was isolated from individual shrimp’s stomach tissue, and was pooled in equal amounts by group for given time points. RNA sequencing was performed on these pools and obtained data was subjected to an analysis of read length distribution frequency. (**a**) Represents read length distribution frequency among total reads obtained for each sample, (**b**) represents read length distribution frequency among reads mapping to a WSSV reference genome, (**c**) represents read length distribution frequency among reads mapping to the VP28 dsRNA treatment sequence, and (**d**) represents the read length distribution frequency among reads mapping to the GFP dsRNA sequence. The Y-axis represents reads per million total reads for a given sample (RPM).

**Figure 9:**
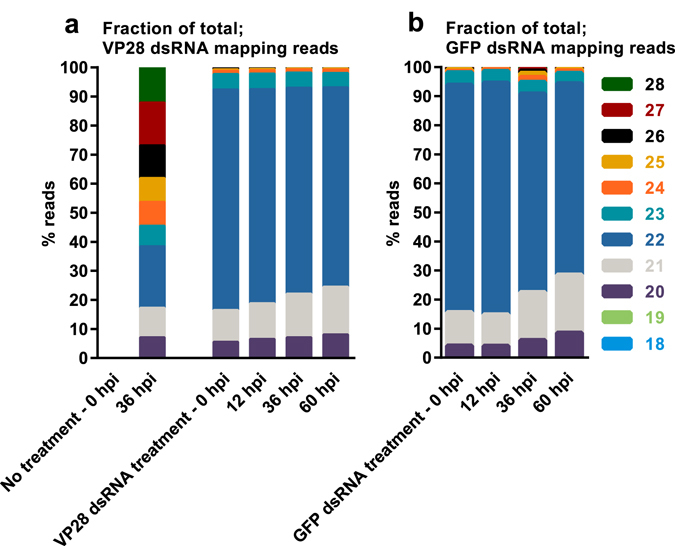
Small RNA length distribution given as fractions of total. RNA was isolated from stomach tissue and equal amounts were pooled for each sample within a given group at a given time point. RNA sequencing was performed on these pools and obtained data was subjected to an analysis of read length distribution frequency. Here, relative distribution of read lengths obtained from reads mapping to the VP28 (**a**) and GFP (**b**) dsRNA treatment sequence are shown to emphasize the dominance of 22 nt reads in samples were the only source of such sequences are the injected dsRNA.

Small RNA libraries were constructed and subsequently sequenced from 5 pooled individuals from each group at each sampling point (exception; VP28 dsRNA group at 60 hpi, n = 4). Mapping of total, trimmed and quality controlled reads to a WSSV reference genome (GenBank accession number KT995471), the VP28 dsRNA sequence and the GFP dsRNA sequence revealed a peak at 22 nucleotide read length in the frequency distribution for all groups (Fig. 8A–D). For the groups receiving dsRNA treatment, the reads mapping to the sequences of their respective dsRNA construct were more narrowly distributed around the center, at 22 nucleotides, in comparison to what was observed for control groups with high WSSV levels where the size-distribution of reads mapping to the full length of the WSSV genome was more widely distributed. Furthermore, fractions of total variation in length frequency distribution revealed that a dominating number of reads in samples where we were certain that the only source for the observed mapping reads was the injected double-stranded RNA, was in fact 22 nucleotides in length (Fig. 9A,B).

### VP28 dsRNA injection generates a siRNA population exclusively targeting gene of interest at high intensity

**Figure 10:**
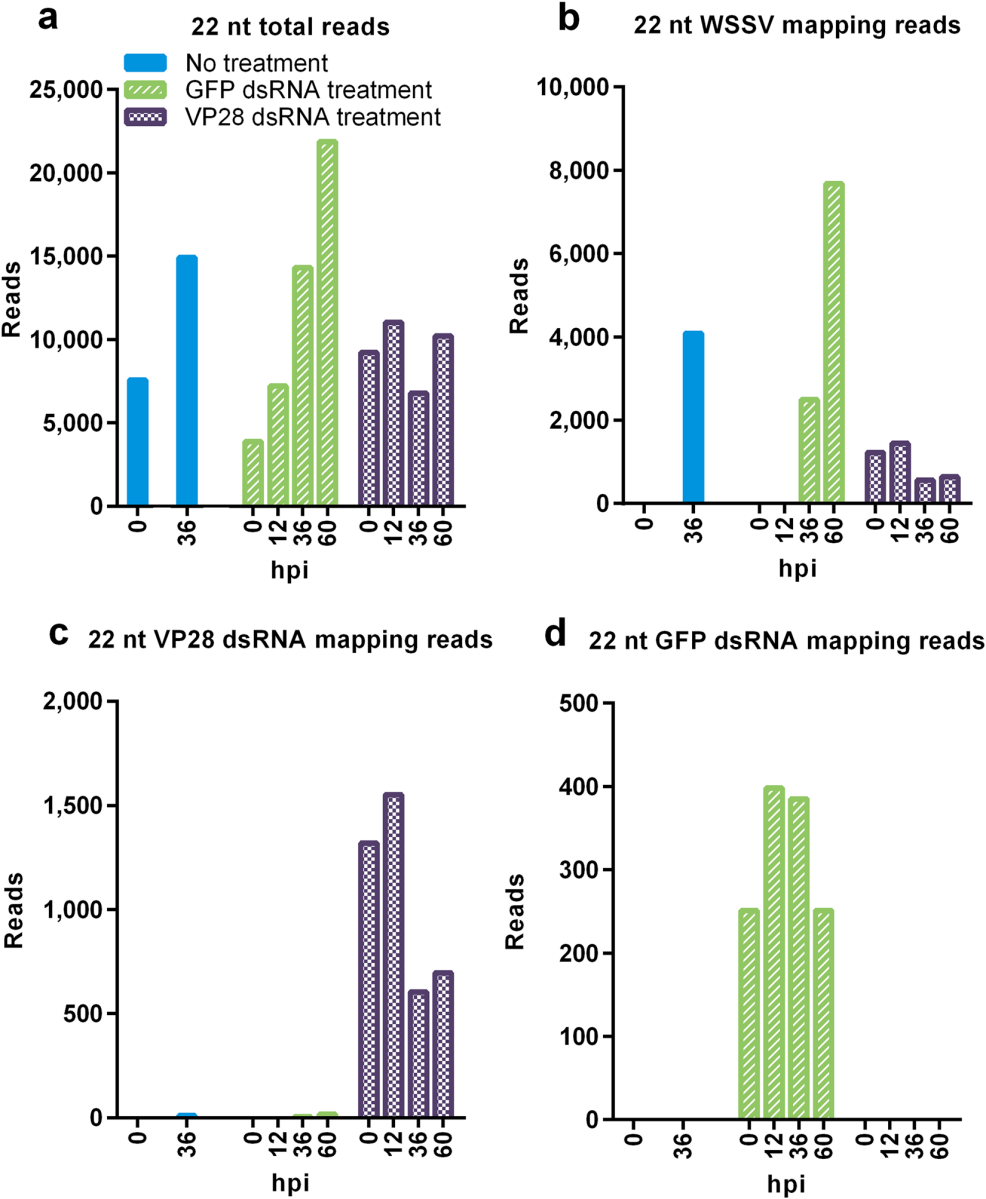
Mapping rate of 22 nt long siRNAs during WSSV infection. RNA was isolated from stomach tissue and equal amounts were pooled for each sample within a given group at a given time point. RNA sequencing was performed on these pools and 22 nt long reads were filtered out to be analyzed separately. (**a**) Represents 22 nt reads frequency among all available reads from each sample, (**b**) represents 22 nt reads frequency among reads mapping to a WSSV reference genome, (**c**) represents 22 nt reads frequency among reads mapping to the VP28 dsRNA treatment sequence, and (**d**) represents the 22 nt reads frequency among reads mapping to the GFP dsRNA sequence. The Y-axis represents reads per million total reads for a given sample (RPM).

**Figure 11:**
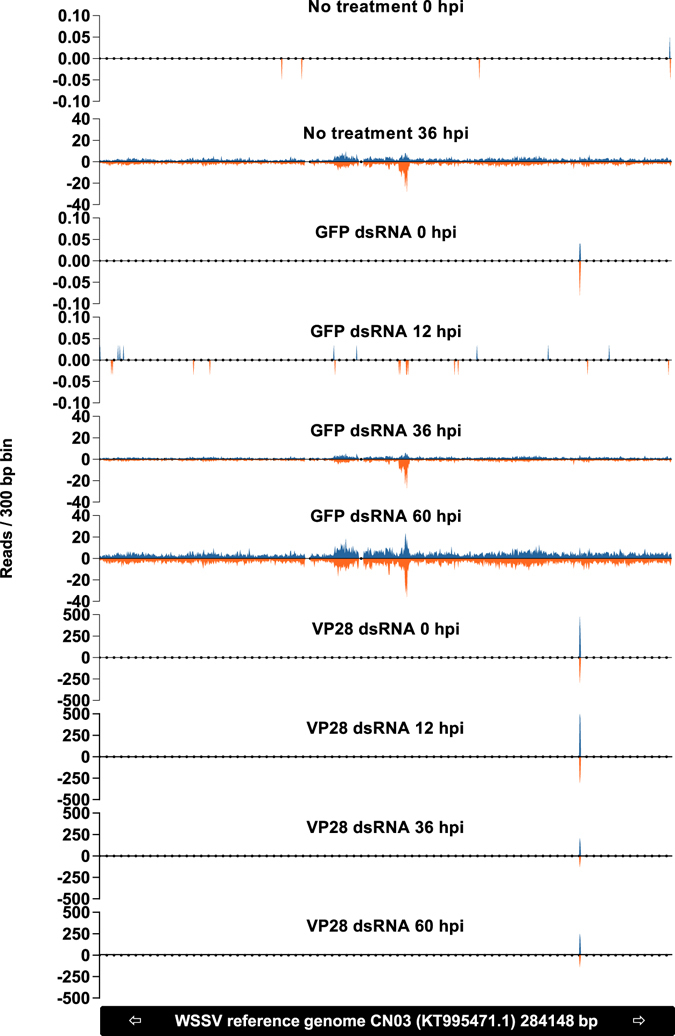
The frequency of 22 nt siRNAs per 300 bp bin across a reference WSSV genome is shown to emphasize the difference between a natural RNAi response to WSSV infection in comparison to a VP28 dsRNA induced siRNA response. RNA was isolated from stomach tissue and equal amounts were pooled for each sample within a given group at a given time point. RNA sequencing was performed on these pools and 22 nt long siRNAs were filtered out to be analyzed separately. The Y-axis represents reads per million total reads for a given sample (RPM), and positive and negative values indicates forward- and reverse mapping siRNAs respectively.

Both the total number of 22 nt long siRNAs and the number of 22 nt siRNAs specifically mapping to a WSSV reference genome (GenBank accession number KT995471) increased consistently in the unprotected control groups as the infection progressed (Fig. 10A,B). In the group protected by VP28 dsRNA treatment, a moderate level of WSSV mapping 22 nt siRNAs was already present prior to infection and remained seemingly stable throughout the sampling period. All 22 nt siRNAs that mapped to the WSSV reference genome in the VP28-treated group mapped to the VP28 dsRNA treatment sequence, indicating that there were no WSSV siRNAs generated in this treatment group as a result of WSSV infection. Only low levels of siRNAs mapping to the VP28-sequence were found throughout the infection period in the unprotected control groups (Fig. 10C). A population of 22 nt siRNAs with specificity for the GFP dsRNA was exclusively generated in the GFP dsRNA treatment group (Fig. 10D). To further illustrate the characteristics of the RNAi response, 22 nt siRNA frequencies for each 300 base pair bin across the WSSV reference genome sequence were plotted for each sample pool (Fig. 11). A gradual, genome-wide, increase in intensity over time was evident for the RNAi response to WSSV infection in the unprotected GFP dsRNA- and PBS control groups. For sample pools where a global siRNA response was evident, namely no treatment control group 36 hpi, nonspecific GFP dsRNA control group 36 and 60 hpi, ROUT method was applied in order to identify outliers at Q = 0.1%. Individual bin frequencies identified as outliers was annotated in respect to position on the WSSV reference genome to identify gene products encoded in these regions. Envelope protein (wsv216), capsid proteins (wsv220, wsv271), E3 ligase (wsv222) and hypothetical proteins (wsv260, wsv277) were found to map the highest coverage of siRNA in all three sample pools. In contrast, the 0 hpi plot for the VP28 dsRNA group revealed an intense and highly focused response within the VP28 dsRNA sequence area already present prior to WSSV infection. This RNAi response pattern remained throughout the sampling period. The ratio between forward mapping siRNAs to reverse mapping siRNAs on the VP28 dsRNA target sequence was found to be 1.75, 1.76, 1.79 and 1.89 respectively for sample pools collected from VP28 dsRNA treatment group at 0, 12, 36 and 60 hpi respectively.

## Discussion

The use of dsRNA to stimulate the RNAi mechanism is receiving attention as a potential tool to protect against viral diseases in shrimp. This study describes how a specific and highly protective RNAi response in *P. vannamei* differs from a nonprotective natural or nonspecific response. Our results show that upregulation of the host genes LvDcr2, LvAgo2 and LvSid-1 occur in both VP28 dsRNA protected shrimp and shrimp injected with a nonprotective and nonspecific GFP dsRNA. This is concerted by the accumulation of a population of 22 nt long siRNAs that specifically targets the sequence of the injected dsRNA. As infection with WSSV progresses, a diverse population of siRNAs targeting the WSSV genome in a global pattern begins to appear in the in unprotected groups, while the population of highly specific siRNAs targeting VP28 remains in the protected group. The slightly lower amount of virus found in the GFP dsRNA control group compared to the PBS injected group suggests that; although the activation of RNAi-associated genes may provide some level of nonspecific protection, the nearly sterilizing effect of injecting VP28-derived dsRNA is mainly contributed to by sequence-specificity. RNA sequencing of small RNAs exclusively originating from two different dsRNA constructs strongly suggests that the dominating cleavage length of LvDcr2 is 22 nucleotides, and thus fully support the proposed idea that Dicer cleaves exogenous dsRNA into a population of 21–23 nt long siRNA effector molecules. Moreover, the intensity of the VP28 dsRNA originating siRNA population and absence of a native siRNA response to WSSV infection, serve to explain the remarkable efficacy obtained by VP28 dsRNA treatment.

Previously, it has been shown that LvDcr2 and LvAgo2 are directly involved in the RNAi response^17, 18^, while LvSid-1 is a putative dsRNA transport molecule potentially involved in uptake and communication of a dsRNA signal between cells^19^. We show how dsRNA induces upregulation of these genes (Fig. 6), although this induction is relatively modest in comparison to expression levels measured after WSSV infection (Fig. 7). Similar results were described by Chen *et al*. (2011), where WSSV infection induced higher expression levels of LvDcr2 compared to the upregulation observed in shrimp treated with Poly (C:G)^18^. Virus specific dsRNA has been shown to provide higher protection in shrimp compared to nonspecific dsRNA^8^, and while the GFP dsRNA treatment of shrimp did not improve survival of shrimp in our study, it was shown that there were significantly lower VP28 mRNA levels at 36 hpi between PBS- and GFP dsRNA control groups. Thus, indicating that GFP dsRNA nonspecific to WSSV does inhibit virus replication, but not to an extent affecting the outcome of the disease in this challenge model.

In a previous small RNA sequencing study on WSSV infected shrimp it was found that small RNAs mapping to WSSV specific protein coding sequences (CDSs) were detected in a higher proportion (>50:1) compared to small RNAs mapping to host CDSs, and that the WSSV specific small RNA population was clearly enriched within the 21–23 nt range. This suggested that that a majority of the WSSV specific small RNA population was not mRNA degradation products, but in fact selectively processed Dicer products^28^. In conjunction with VP28 mRNA not being detected in the VP28 dsRNA treatment group during challenge, we deduced that the injected VP28 dsRNA was the only source that could be processed into the VP28 dsRNA mapping small RNAs. The VP28 dsRNA mapping small RNAs were the only WSSV mapping reads in these samples, and the size distribution of these reads shows that the overall majority of reads is 22 nucleotides in length. Similarly, reads mapping to the nonspecific GFP dsRNA sequence could only be found in shrimp that had been injected with this control treatment, and the same read length profile was evident for these reads as well. Thus, in *P. vannamei*, dsRNA is predominantly cleaved into short double strands consisting of 22-nucleotide short RNAs, and this suggests, more specifically, that LvDcr2 cleaves dsRNA into siRNA duplexes mainly consisting of 22-nucleotide RNAs.

When the distribution of siRNAs across the WSSV reference genome was examined between VP28 dsRNA treatment group and the control groups, the difference was remarkable. In the PBS- and the GFP dsRNA control groups a siRNA population with relatively low coverage and broad distribution developed as WSSV infection progressed. For the nonspecific GFP dsRNA control group at 60 hpi, which exhibited the strongest RNAi response observed among controls, a peak coverage of 36 occurred in bin starting at WSSV genome position 152701 (wsv277) and an average coverage of 4.14 RPM per bin (RPM = reads per million total reads in sample) was calculated across the reference genome. In contrast, the observed siRNA population in the VP28 specific dsRNA treatment group was restricted to the bins spanning the genome region where the VP28 dsRNA sequence is found [238501–239100], exhibiting peak coverage of 502 RPM and average siRNA coverage of 371 RPM within these bins at 12 hpi. The ratio between forward- and reverse strand mapping siRNAs aligning to the target sequence injected into the VP28 specific dsRNA group was consistent over time. VP28 is encoded on the WSSV forward strand, and, since there was more than 1.75 forward strand mapping siRNAs for every reverse strand mapping siRNA, the lower proportion of siRNAs would actually be targeting VP28 mRNAs transcribed after WSSV challenge. Thus, although the RNAi mechanism was significantly higher upregulated and producing a higher overall number of siRNAs mapping to the WSSV reference genome for the control groups, the targeted knockdown of VP28 through a low-level RNAi induction in the VP28 dsRNA treatment group proved to be more beneficial in regards to the observed impact on virus replication and survival. WSSV replication rate and siRNA coverage in control groups suggest that the native RNAi response occurs too late with a too general siRNA distribution of insufficient coverage. In *Drosophila*, flies with functional Dcr-2 infected with invertebrate iridescent virus 6 (IIV-6) had higher survival in comparison to observed mortality in Dcr-2 mutant flies. Interestingly, the wild type flies’ relatively higher ability to cope with the virus infection coincided with a virus specific siRNA population characterized by pronounced hotspots, suggesting that such focusing of the siRNA response could be beneficial for the host^30^. In our WSSV siRNA analysis, we did not observe siRNA hotspots to the same extent as in IIV-6 infected *Drosophila*, although a small subset of genes was identified by a stringent outlier detection method. Among these genes we identified siRNA hotspots within wsv271 and wsv277 in no treatment control group at 36 hpi and in nonspecific GFP dsRNA control group at 36 and 60 hpi. These hotspots have previously been reported, in addition to a hotspot in wsv360^28^. However, we could only identify wsv360 as a hotspot in the nonspecific GFP dsRNA control group at 60 hpi, which potentially emphasize a time-dependent variation in specific siRNA levels.

We conclude that, in response to a WSSV VP28 specific dsRNA treatment, a relatively modest induction of the RNAi siRNA-pathway leads to a highly intensive and focused siRNA population that inhibits WSSV replication and WSSV-induced mortality. To our knowledge this is the first time the siRNA population after dsRNA treatment has been characterized to this extent.

## Material and Methods

### dsRNA treatment

Two different dsRNA constructs, VP28 dsRNA (600 base pairs) and GFP dsRNA (240 bp), were procured from agroRNA (Seoul, Korea) and delivered in a proprietary dsRNA transcription solution. The respective nucleotide sequences are listed under Supplementary Data S1. dsRNA was diluted in sterile PBS to a final concentration of 0.857 mg/ml to be administered in a 0.05 ml IM dose per shrimp. Based on a sampled weight distribution among reservoir shrimp (n = 30), this dose ensured that shrimp smaller or equal to the registered max weight at 3.57 g ([1.8, 3.57], mean = 2.62 g) would receive a dsRNA dose ≥12 µg dsRNA/g shrimp body weight.

### WSSV challenge isolate

Samples were collected from WSD affected *P. vannamei* on a shrimp farm in Loc An, Vung Tau Province, Vietnam in January 2015 by MINH PHU AQUAMEKONG SHRIMPVET LABORATORY and given the case study number 15002. WSSV was confirmed by qPCR detection in multiple samples (results not shown). Both WSSV positive- and WSSV negative challenge inoculums were prepared. Shrimp (*P. vannamei*) either positive- or negative for WSSV, stored at −80 °C, were thawed and cuticle was removed. In separate procedures for either WSSV positive or –negative tissue, the shrimp tissues were homogenized (10% w/v) in sterile 0.09% NaCl buffer, spun at 2000 rpm for 10 min and finally filtered (0.45 µm) before preparing aliquots stored at −80 °C. This challenge stock was referred to as a 1:10 dilution.

### dsRNA treatment homology to challenge isolate

RNA sequence reads, with maximum three mismatches outside a seed length of 10, mapping to WSSV genome (GenBank accession number KT995472) obtained from a small RNA sequence library, consisting of a pool of 5 individual shrimp RNA samples from the no treatment control group 36 hpi with WSSV, was de novo assembled into predicted transcripts using Velvet and Oases. Predicted WSSV transcripts were subjected to BLASTn with the different dsRNA treatment sequences as query, and predicted transcripts with hits were assembled in CLC Main Workbench to achieve a consensus sequence. The consensus sequence was then subjected to another BLASTn with the VP28 dsRNA treatment sequences as query, and results showed 598/600 identities and no gaps. There was no match between predicted WSSV transcripts and the GFP dsRNA. Thus, VP28 dsRNA treatment sequence was specific in regards to the WSSV challenge isolate (with two mismatching bases), and the GFP dsRNA treatment sequence was indeed nonspecific in regards to the WSSV challenge isolate.

### WSSV challenge trial

*In vivo* shrimp trials were performed at MINH PHU AQUAMEKONG SHRIMPVET LABORATORY in Viet Nam, a national reference laboratory for diagnostics and surveillance of shrimp pathogens, under a general approval for experiments with live decapod crustaceans from the Vietnamese Ministry of Agriculture. Although there are no official guidelines for conducting experiments on live decapod crustaceans in Viet Nam, the experiments described herein were conducted according to the principle of the 3Rs and termination at a humane endpoint. Through several pre-challenge trials (results not shown), a suitable challenge dose, resulting in >90% accumulated mortality within 72 hours, was found to be 0.05 ml 1E-4 WSSV inoculum dilution (in PBS) per shrimp. By absolute quantification by qPCR the challenge dose was estimated to contain ~1000 WSSV genome copies (results not shown). In the challenge trial each group consisted of 100 study animals (*P. vannamei*, sampled mean weight; 2.618 g ± 0.527 SD, n = 30) divided equally between five 120 L tanks, held in a temperature controlled biosecure facility (28 ± 1 °C), containing brackish water with 20 ppt salinity, set up with individual biofilters and aeration. Treatments were administered simultaneously with tank allocation, and the shrimp were challenged 48 hours after treatment administration. Following administration of challenge inoculum tanks were monitored 4 times per day, including recording and removal of mortalities and feeding according to appetite. One tank per group acted as sampling unit for live samples collected at 0, 12, 36 and 60 hpi. Five individual shrimp were sampled from each group at 0, 12, 36 and 60 hpi, with the exceptions; PBS control group only sampled at 0 and at 36 hpi, and reduced sample in VP28 specific dsRNA treatment group at 60 hpi (n = 4).

### RNA and DNA isolation

RNA for RNA sequencing and RT-qPCR was isolated individually from shrimp proventriculus (stomach) tissue collected at different time points with Direct-zol^TM^ RNA MiniPrep Plus w/TRI Reagent® (Zymo Research) according to manufacturer’s guidelines. RNA concentration was measured with a Picodrop spectrophotometer. Picodrop measurements were conducted on both undiluted and 1:10 diluted RNA to check uniformity in measurements across dilutions, and the average between the two obtained concentrations was used as input in normalizing RNA concentration between individual samples. RNA quality (RIN) in RNA pools subjected to RNA sequencing was determined on Agilent’s BioAnalyzer. DNA for qPCR was isolated from shrimp uropod tissue with a ZR Viral DNA Kit^TM^ (Zymo Research) according to manufacturer’s guidelines.

### PCR

Isolated DNA from individual uropod samples was diluted 1:50 and 5 µl was added to a reaction mix containing 6 µl 2X SYBR Select Master Mix (Life Technologies/Applied Biosystems), 0.5 µl 3.6 µM VP28QF (5′-GGGAACATTCAAGGTGTGGA-3′) primer^31^ and 0.5 µl 3.6 µM VP28QR (5′-GGTGAAGGAGGAGGTGTTGG-3′) primer^31^. In addition to isolated DNA from shrimp samples, each plate included a standard curve composed by a 10-fold dilution series of a plasmid containing a WSSV VP28 insertion and an internal plate calibrator sample. Each DNA sample and plasmid standard dilution was run in triplicate wells on an Agilent AriaMx Realtime PCR System with the following cycling conditions; 50 °C–2 min, 95 °C–2 min, [95 °C–15 s, 60 °C–20 s, 72 °C–10 s] × 40 cycles ending with a melt curve analysis.

A two-step RT-qPCR procedure was applied on individual RNA samples, in which 14 µl 125 ng/µl RNA was added to 20 µl final volume RT reaction using SuperScript® VILO™ cDNA Synthesis Kit (ThermoFisher/Invitrogen) according to manufacturer’s guidelines (25 °C–10 min, 42 °C–120 min, 85 °C–5 min). cDNA from individual samples was diluted 1:500 and 5 µl was added to a reaction mix containing 6 µl 2X SYBR Select Master Mix (Life Technologies/Applied Biosystems), 0.5 µl forward primer and 0.5 µl 3.6 µM reverse primer. In addition to cDNA from shrimp samples, each plate included an internal plate calibrator sample. For each individual template 9 different PCR assays were included; LvDcr1F (5′-CCGGAGATAGAACGGTTCAGTG-3′)^21^/LvDcr1R (5′-CGATAATTCCTCCCAACACCTG-3′)^21^, LvDcr2F (5′-AGGAAATGCAATGTCGTGGTT-3′)^18^/LvDcr2R (5′-ACGAGCCCTCCCCCTAGATT-3′)^18^, LvAgo1F (5′-TGCGTCATTTGCCATCCAT-3′)^19^/LvAgo1R (5′-GCCATCTGGAGCGGAGAAG-3′)^19^, LvAgo2F (5′-GATGGCATGAAGTCTGCAGTTG-3′)^19^/LvAgo2R (5′-TGCGCACGACCATCACTAAG-3′)^19^, LvSid-1F (5′-GAAGCGATTGGCAGTCTATGAAC-3′)^19^/LvSid-1R (5′-TGGAAGCCTATCTCTGCAACTTG-3′)^19^, VP28QF/VP28QR assay annealing within the boundaries set by the sequence comprising the WSSV VP28 dsRNA treatment, VP28Q2F (5′-ACATCACTGGTATGCAGATGGT-3′)/VP28Q3R (5′-AGCACGATTTATTTACTCGGTCTC-3′) assay of which the reverse primer anneals to the end of WSSV VP28 ORF and start of the 3′ UTR thus amplifying WSSV VP28 mRNA without specificity for the WSSV VP28 dsRNA treatment, b-actinQF (5′-GAACCTCTCGTTGCCGATGGTG-3′)^31^/b-actinQ2R (5′-GAAACTGTGCTACGTGGCCCTG-3′) assay for quantification of reference gene #1 and LvRpS3Afwd (5′-GGCTTGCTATGGTGTGCTCC-3′)^32^/LvRpS3Arev (5′-TCATGCTCTTGGCTCGCTG-3′)^32^ assay as quantification of reference gene #2. For each assay, each cDNA sample was run in triplicate wells on an Agilent AriaMx Realtime PCR System with the following cycling conditions; 50 °C–2 min, 95 °C–2 min, [95 °C–15 s, 60 °C–20 s, 72 °C–10 s] × 40 cycles ending with a melt curve analysis. qPCR primers were tested with an endpoint PCR setup to confirm specificity of primers (results not shown). For each individual sample, Cq-value outliers, if any, among the triplicates were systematically removed for each assay if one value did show a >0.5 deviation from the mean Cq of the two most similar values (in any direction). For each assay, normalized expression (NE) with kinetic PCR efficiency (E) correction was calculated for each individual sample with the mean of the Cq-values obtained from the two listed references genes used as normalizing factor^33^. When “No Cq”-results were obtained, Cq-value = 40 was used in relative quantification. The lowest NE value obtained for a particular assay was used as divisor for all samples’ NE value to achieve relative fold variation in gene expression between samples.

### RNA sequencing

Small RNA sequencing was performed on pools of 5 individual RNA samples from each group sampled at 0, 12, 36 and 60 hpi, with the exceptions; PBS control group only sampled at 0 and 36 hpi and reduced sample in VP28 specific dsRNA treatment group at 60 hpi (n = 4). Each individual RNA sample contributed with an equal amount of total RNA mass per pool based on Picodrop measurements, and each pool was aimed to end up with a final RNA concentration of 200 ng/µl, although a repeated measurement performed on prepared pools did show the average RNA concentration to be 195 ng/µl ± 11 SD (ng/µl range [173, 208]). Small RNA sequencing was performed at Theragenetex (Seoul, Korea), and consisted of RNA sample quality control (Bioanalyzer), library preparation with a TruSeq® Small RNA Library Prep Kit (Illumina) and 50 SE sequencing with 20 M reads per sample on a HiSeq2500 rapid run platform (Illumina). The sequencing was performed according to Illumina’s standard ISO protocol.

### Bioinformatics

Raw data was obtained from the sequencing service provided by Theragenetex (Seoul, Korea). Adaptor sequence (TGGAATTCTCGGGTGCCAAGG) was removed from reads in the TruSeq® Small RNA libraries using cutadapt v1.9.1^34^, and adaptor-trimmed reads were processed through sickle^35^ (options; sickle se -t sanger -q 20) for removal of low quality reads. Cutadapt v1.9.1 was also used in order to remove reads <22 and reads >22 using options; cutadapt -m 22 -M 22.

Mapping of reads was performed using bowtie1 v1.1.2^36^, in which bowtie-build was used to construct index files for the VP28 and GFP dsRNA treatment sequences in addition to the WSSV reference genome sequence (GenBank accession number KT995471). For creating alignments used in analysis of 22 nt coverage and distribution across the reference genome, Bowtie1 was run with the following options; -q -v 0–sam and was performed for both strands in the reference sequence using–norc and–nofw. These options did not allow for any mismatches in the alignment. Sam files were converted to.bam files using samtools view, and samtools stats was applied to obtain read length frequency distribution for each alignment (Samtools v1.3.1^37^).

For filtering out reads (total reads as input) aligning to the WSSV reference genome subsequently to be used in de novo contig assembly, Bowtie1 was run with options; -q -n 3 -l 10 –sam. WSSV aligning reads were assembled into contigs for each of the k-mer lengths 15, 17, 19, 21 and 23 using velvet v1.2.10^38^ (options; -sam -short -cov_cutoff 5 -read_trkg yes). Contigs were assembled into predicted transcripts using Oases^39^. In the presented data, read counts are normalized to million total reads obtained for the respective sample (RPM = reads per million).

### Statistics

Log-rank (Mantel-Cox) test with Bonferroni correction was applied when testing for statistical significance at p = 0.05 between groups in the challenge trial. Bonferroni correction was carried out by dividing the α-level (0.05) with the total number of possible pairwise comparisons included in the particular study. One-way ANOVA with Dunnett’s, Bonferroni’s or Tukey’s post hoc test was applied in order to identify statistical significance at p = 0.05 between groups in qPCR studies. Dunnett’s post hoc test was applied when comparing groups to a single base line level group. Bonferroni’s post hoc test was applied when conducting a single pair-wise comparison between two groups in a study, while Tukey’s post hoc test was applied when performing pairwise comparisons between all groups in a single study’s subfamily. All statistical analyses were conducted in GraphPad Prism v6.07.

## Electronic supplementary material

Supplementary Data S1
