# Supplementary material for: White spot syndrome virus VP28 specific double-stranded RNA provides protection through a highly focused siRNA population
Source: Sci Rep. 2017 Apr 21;7:1028. doi: 10.1038/s41598-017-01181-w (PMC5430881; doi:10.1038/s41598-017-01181-w)
Supplement: Supplementary file 1 — Supplementary Data S1 [file 41598_2017_1181_MOESM1_ESM.pdf]

White spot syndrome virus VP28 specific double-stranded RNA provides protection through a highly focused siRNA population

Pål Nilsen, Marius Karlsen, Kallaya Sritunyalucksana, Siripong Thitamadee

Supplementary Data S1

dsRNA treatment sequences

GFP

Length; 240bp

Sequence;

ATGGTGAGCAAGGGCGAGGAGCTGTTCACCGGGGTGGTGCCCATCCTGGTCGAGCTGGACGGC
GACGTAAACGGCCACAAGTTCAGCGTGTCCGGCGAGGGCGAGGGCGATGCCACCTACGGCAAG
CTGACCCTGAAGTTCATCTGCACCACCGGCAAGCTGCCCGTGCCCTGGCCCACCCTCGTGACCA
CCCTGACCTACGGCGTGCAGTGCTTCAGCCGCTACCCCGACCACATGAAG

VP28

Length; 600bp

Sequence;

ATGGATCTTTCTTTCACTCTTTCGGTCGTGTCGGCCATCCTCGCCATCACTGCTGTGATTGCTGTA
TTTATTGTGATTTTTAGGTATCACAACACTGTGACCAAGACCATCGAAACCCACACAGACAATATC
GAGACAAACATGGATGAAAACCTCCGCATTCCTGTGACTGCTGAGGTTGGATCAGGCTACTTCAA
GATGACTGATGTGTCCTTTGACAGCGACACCTTGGGCAAAATCAAGATCCGCAATGGAAAGTCTG
ATGCACAGATGAAGGAAGAAGATGCGGATCTTGTCATCACTCCCGTGGAGGGCCGAGCACTCGA
AGTGACTGTGGGGCAGAATCTCACCTTTGAGGGAACATTCAAGGTGTGGAACAACACATCAAGAA
AGATCAACATCGCTGGTATGCAGATGGTGCCAAAGATTAACCCATCAAAGGCCTTTGTCGGTAGC
TCCAACACCTCCTCCTTCACCCCCGTCTCTATTGATGAGGATGAAGTTGGCACCTTTGTGTGTGG
TACCACCTTTGGCGCACCAATTGCAGCTACCGCCGGTGGAAATCTTTTCGACATGTACGTGCACG
TCACCTACTCTGGC
